# Antibiotic Prophylaxis Prior to Dental Procedures

**DOI:** 10.3390/dj12110364

**Published:** 2024-11-15

**Authors:** Danica Vidović Juras, Ivana Škrinjar, Tena Križnik, Ana Andabak Rogulj, Božana Lončar Brzak, Dragana Gabrić, Marko Granić, Kristina Peroš, Ivana Šutej, Ana Ivanišević

**Affiliations:** 1 Department of Oral Medicine, School of Dental Medicine, University of Zagreb, Gunduliceva 5, 10000 Zagreb, Croatia; djuras@sfzg.unizg.hr (D.V.J.); andabak@sfzg.unizg.hr (A.A.R.); loncar@sfzg.unizg.hr (B.L.B.); 2 Department of Oral Medicine, Dental Clinic, Clinical Hospital Centre Zagreb, Gunduliceva 5, 10000 Zagreb, Croatia; 3 Community Health Centre, Mirka Crkvenca 1, 49000 Krapina, Croatia; tena.kriznik0210@gmail.com; 4 Department of Oral Surgery, School of Dental Medicine, University of Zagreb, Gunduliceva 5, 10000 Zagreb, Croatia; dgabric@sfzg.unizg.hr (D.G.); granic@sfzg.unizg.hr (M.G.); 5 Department of Oral Surgery, Dental Clinic, Clinical Hospital Centre Zagreb, Gunduliceva 5, 10000 Zagreb, Croatia; 6 Department of Pharmacology, School of Dental Medicine, University of Zagreb, Salata 3b, 10000 Zagreb, Croatia; peros@sfzg.unizg.hr (K.P.); isutej@sfzg.unizg.hr (I.Š.); 7 Department of Endodontics and Restorative Dentistry, School of Dental Medicine, University of Zagreb, Gunduliceva 5, 10000 Zagreb, Croatia; aivanisevic@sfzg.unizg.hr; 8 Department of Endodontics and Restorative Dentistry, Dental Clinic, Clinical Hospital Centre Zagreb, Gunduliceva 5, 10000 Zagreb, Croatia

**Keywords:** antibiotic prophylaxis, antibiotic resistance, dental procedures

## Abstract

Antibiotic prophylaxis in dentistry has been recommended for different groups of patients, such as patients with impaired immunologic function, patients at risk of developing infective endocarditis or prosthetic joint infection, patients previously exposed to high-dose irradiation of the head and neck regions, and patients receiving intravenous bisphosphonate and antiangiogenic treatment. The guidelines have been changed over the years, and the list of medical conditions requiring antibiotic prophylaxis has been shortened considerably in the context of antibiotic resistance and unnecessary antibiotic prescription.

## 1. Introduction

Bacteremia presents the presence of bacteria in the bloodstream. Transient bacteremia is associated with invasive dental procedures but it can also occur after everyday activities like chewing, flossing, and teeth brushing, and in healthy individuals it usually does not cause the development of symptoms. However, in some patients, bacteremia can lead to serious complications [1].

Antibiotic prophylaxis (AP) is defined as a procedure of prescribing antibiotics to prevent the development of a bacterial infection. Antibiotic prophylaxis in dentistry usually involves a single dose of antibiotics 30 min to 1 h before an invasive dental procedure, and its effectiveness is short-lived, limited to several hours after the procedure during transient bacteremia. Antibiotic prophylaxis is directed at multiple pathogens to achieve an adequate antimicrobial effect by reducing their number or completely eliminating them from the bloodstream [2].

Recommendations for antibiotic prophylaxis before dental procedures were first designed back in 1955 [1]. Antibiotic prophylaxis in dentistry has been recommended for different groups of patients as patients with a damaged immune system, patients who are at risk of developing infective endocarditis (IE) or prosthetic joint infection after a dental procedure, patients previously exposed to high-dose irradiation of the head and neck regions, and patients receiving intravenous bisphosphonate and antiangiogenic treatment. The guidelines have been changed over the years, and the list of medical conditions requiring antibiotic prophylaxis was considerably shortened in the context of antibiotic resistance and unnecessary antibiotic prescribing. The continuous updating of the guidelines is a necessary consequence of new findings.

The most common drugs prescribed for antibiotic prophylaxis in dentistry are antibiotics from the β-lactam group. The recommended first choice of antibiotic for the patients not allergic to penicillin is oral amoxicillin, and, if the person is not able to take the drug orally due to a certain condition, ampicillin is administered parenterally. Cephalosporins are the second choice, but only if a patient had no history of a real and severe allergic reaction to penicillin, due to a possible cross-reaction between cephalosporins and penicillin [3]. If a person is allergic to penicillin, azithromycin, or clarithromycin should be prescribed [4].

In 2021, the American Heart Association (AHA) recommended that clindamycin should no longer be used for the prevention of IE in the case of allergies to amoxicillin or ampicillin because clindamycin “may cause more frequent and severe adverse reactions than other antibiotics used for antibiotic prophylaxis” (this also includes infection with *C. difficile*) [4].

According to the World Health Organization (WHO), the emergence of resistance to antibiotics is accelerating. The main reasons for this are the misuse and overuse of antibiotics, and the insufficient prevention and control of infections. Antibiotic resistance will remain a great problem, even if we develop new drugs, if we do not change the way antibiotics are prescribed [5]. Infection with antimicrobial resistant bacteria can cause a prolonged stay in hospital due to serious illnesses. This leads to higher costs for the healthcare system, worse outcomes, and treatment failures [6]. According to a study published in The Lancet in 2019, in the period between 2007 and 2015, the number of deaths caused by antibiotic resistance in EU/EEA countries increased by 2.5 times. The number of deaths due to infections with *C. pneumoniae* resistant to carbapenems increased the most (6.16 times). The results of the study in The Lancet showed that the burden of infections caused by bacteria that are resistant to antibiotics is higher in infants. In the adult population, these infections increase with age. As the EU/EE population is getting older, this could result in the increased problem of infections caused by antibiotic-resistant bacteria [7].

The aim of this narrative review was to present the contemporary principles and guidelines for antibiotic prophylaxis, and to emphasize the importance of rational prescription of antibiotics due to the increasing development of antibiotic resistance as an important global problem.

## 2. Antibiotic Prophylaxis Prior to Dental Procedure Depending on the Indication

There are a number of medical conditions where antibiotic prophylaxis prior to an invasive dental procedure may be considered. These include patients with the risk for the development of infective endocarditis, patients with artificial joints, patients with diabetes, patients with transplanted organs, patients at risk of osteoradionecrosis of the jaw (previous head and neck irradiation), patients at risk of medically related osteonecrosis of the jaw (MRONJ) (patients taking antiresorptive and antiangiogenic drugs), patients with HIV infection, patients on dialysis, and patients on biological therapy.

### 2.1. Antibiotic Prophylaxis in a Patient with a Risk for IE Development

Based on a comprehensive review of papers published in the relevant literature, AHA adopted the new guidelines for the prevention of IE before dental procedures in May 2021. Despite the fact that the disease is rare, and the fact that there is no convincing direct evidence that dental procedures induce IE, the AHA continues to recommend antibiotic prophylaxis prior to dental procedures in some cases [4]. The AHA also considers that prophylaxis before dental procedures can only prevent a small percentage of IE, even if prophylaxis would be completely effective [4,8].

According to the 2021 AHA guidelines, antibiotic prophylaxis is indicated before certain dental procedures in groups of cardiovascular patients who are at greater risk of poor outcome from IE [8,9]. Prior to a dental procedure which involves an operation in the gingival area, periapical tooth manipulation and biopsy/perforation or oral mucosal tissue antibiotic prophylaxis should be prescribed in the group of patients with valvular heart disease and any of the following diagnoses listed in Table 1:

Prescribing AP in patients after aortocoronary bypass surgery or following the implantation of coronary stents in arteries, electronic devices such as pacemakers or defibrillators, or ventriculoatrial shunts in the central nervous system, is not necessary [4].

Current American Dental Association (ADA) recommendations available online at the time of writing this paper (August 2023) recommend doctors of dental medicine to follow 2021 AHA recommendations [10].

In addition, they emphasize that, for the prevention of IE, good oral hygiene and regular dental examinations every 6 months are crucial. Antibiotic prophylaxis is recommended for every dental procedure which involves the gingival tissue, the periapical tooth area, or biopsy/perforation of the oral mucosal tissue [4].

Antibiotic prophylaxis should not be used in the case of [4,8]:

- the application of anesthetic in non-inflamed tissue;
- taking dental X-rays;
- during the placement of mobile prosthetic and orthodontic devices;
- adjustments to orthodontic appliances;
- the placement of orthodontic braces;
- the falling out of deciduous teeth and lips, or oral mucosa bleeding due to trauma.

Antibiotic prophylaxis regimens for dental procedures according to AHA [4] include oral Amoxicillin in doses of 2 g or 50 mg/kg in children. If the patient is unable to take oral medication, intramuscular (IM) or intravenous (IV) Ampicillin in a dose of 2 g should be used in adults, and 50 mg/kg in children. Alternative medication is Cefazolin or Ceftriaxsone in a dose of 1 g IM or IV in adults, and 50 mg/kg in children. If the patient is allergic to penicillin or ampicillin, oral Cephalexin in a dose of 2 g in adults and 50 mg/kg in children should be used (or other first- or second-generation oral cephalosporin equivalents for adults or children). An alternative medication is oral Azithromycin or Clarithromycin in a dose of 500 mg in adults and 15 mg/kg in children, or Doxycycline in a dose of 100 mg in adults and 2.2 mg/kg (<45 kg) or 100 mg (>45 kg). If the patient is allergic to penicillin or ampicillin, and unable to take oral medication, Cefazolin or Ceftriaxone in a dose of 1 g IM or IV, and 50 mg/kg in children, is indicated (in case of a history of anaphylaxis, angioedema, or urticaria to penicillin, Cephalosporins should not be used).

Prophylaxis should be taken in one dose, 30–60 min before the procedure. If prophylaxis is not given before the procedure, it can be given afterwards within 2 h. In patients who are already taking a full dose of antibiotics (administered for 7–10 days) which are recommended for antibiotic prophylaxis, it is necessary to administer another of the recommended antibiotics if the last dose of antibiotic was administered more than 3 h ago. Alternatively, the procedure can be postponed for 10 days after the last dose of antibiotics [4,9]. Patients who are on intravenous antimicrobial therapy due to IE or some other infection, and require a specific invasive dental procedure, still receive the applied antibiotic parenterally during the procedure [4].

During dental procedures that last longer than 6 h, it is necessary to repeat the prophylaxis with the same doses [11].

Recently, the European Society of Cardiology (ESC) has published updated guidelines which are in accordance with AHA guidelines [12].

### 2.2. Antibiotic Prophylaxis in Patients with Artificial Joints

In 2015, the Scientific Committee of the ADA concluded, based on a review of the literature, and at the same time considering clinical recommendations from 2012 and 2013, that there is no objective proven association between invasive dental procedures and the infection of artificial joints.

Accordingly, the Committee recommended that routine antibiotic prophylaxis in patients with artificial joints is not necessary. If a dentist considers the prescription of antibiotic prophylaxis due to a certain complex condition, for example in the case of a previous infection of the artificial joint, it is best that an orthopedic surgeon assess the need for prophylaxis and prescribe the best antibiotic [13].

European guidelines for prescribing antibiotic prophylaxis in patients with artificial joints are adopted on the basis of an extensive review of the literature. The working group concluded in 2017 that it is not necessary to prescribe antibiotic prophylaxis before invasive dental procedures, even in immunocompromised patients. Emphasizing the importance of oral hygiene and regular dental check-ups are the most important preventive measures and can minimize the risk of infection of the artificial joint during dental procedures [14].

### 2.3. Antibiotic Prophylaxis in Patients with Diabetes

By reviewing the literature on prescribing antibiotic prophylaxis in patients with diabetes before invasive procedures, it was concluded that there are no scientifically based facts that would speak in favor of an increased risk of postoperative infections, especially if patients have normal glycemic control. Therefore, antibiotic prophylaxis should not be routinely prescribed to patients with diabetes [15].

In patients with diabetes, prophylactic antibiotics are not generally required. They may be given in postoperative setting for patients with very difficult to control diabetes if the invasive procedure is performed. The need for antibiotics in these situations is also indicated if the fasting plasma glucose levels exceed 200 mg/dL. If the clinical response is poor, it is best to prescribe a more effective antibiotic based on antibiotic sensitivity results [16], as shown in Table 2.

### 2.4. Antibiotic Prophylaxis in Patients with Transplanted Organs

The approach to the dental treatment of patients with transplanted organs can be divided into two periods, pre-transplantation and post-transplantation. Consultation with a patient’s doctor is essential in each case. In the pre-transplantation period, it is necessary to remove all potential factors that could lead to infection in the oral cavity in the post-transplantation period. Recent studies support the influence of local intraoral infections on the prognosis of a transplanted organ [17].

Searching literature, there is no generally accepted, strictly prescribed protocol regarding the use of antibiotic prophylaxis (or antibiotic therapy) in the pre-transplantation period, but antibiotic prophylaxis can be justified since patients may have an uncontrolled systemic disease such as uncontrolled diabetes, high-risk cardiovascular diseases, blood dyscrasias, and others [18].

Patients with leukemia or lymphoma undergoing a stem cell transplantation may be prone to infection due to neutropenia. Before an invasive dental procedure, a dentist should have an insight into laboratory findings like complete blood count, coagulation test, and others, depending on the underlying disease.

In the post-transplantation period, patients are at high risk of infection due to a high dose of immunosuppressants, and it is justified to prescribe antibiotic prophylaxis.

There are no universally agreed-upon indications for antibiotic prophylaxis before dental treatment due to immunosuppression alone [19].

Antibiotic prophylaxis is usually prescribed according to the AHA guidelines for the prevention of IE in patients with transplanted heart who develop valvular heart disease. [4]. The American Academy of Pediatric Dentists (AAPD) guidelines recommend considering prescribing prophylactic antibiotics according to the AHA guidelines for pediatric dental patients at risk for infection, including transplant recipients on immunosuppressive therapy when ANC (absolute neutrophile count) is between 1000 and 2000 cells/mm^3^; when the ANC is <1000, the AAPD recommends antibiotic prophylaxis of an extended course of antibiotic [20,21].

Recommendations from the literature are summarized in Table 3.

However, the fact that immunosuppressive drugs change the oral microflora should be taken into account, and it is difficult to determine the most suitable antibiotic for prophylaxis. There should be an individual approach to each patient, including a consultation with the patient’s doctor. If the oral hygiene is satisfactory, and the graft is stable, prophylaxis is not even necessary. However, if the patient is taking high doses of immunosuppressants and has active periodontitis, the risk of infection is high, and AP should be considered. In each case, consultation with physicians is necessary.

Although there is no universal algorithm for patients who are preparing for transplantation or those in the post-transplantation period, at our University Dental Clinic we follow AHA and AAPD guidelines, closely co-operating with the patient’s transplant team.

### 2.5. Antibiotic Prophylaxis in Patients at Risk of Osteonecrosis of the Jaw

#### 2.5.1. Patients Who Had Head and Neck Irradiation Therapy

In patients who have undergone head and neck irradiation, the blood flow through the bone is reduced, and it is questionable whether the antibiotic can reach the affected part of the bone [22]. Despite this, AP is recommended for patients irradiated in the head and neck areas in order to prevent osteoradionecrosis of the jaw. In addition, most authors believe that the injury of the oral mucosa represents the entry point for oral microorganisms to the irradiated part of the jaw [23].

Antibiotic prescription regimens for these patients during invasive dental procedures vary greatly between clinics and clinicians. No universally accepted guidelines exist. Al-Bazie et al. [23] prescribe amoxicillin in a dose of 500 mg, or clindamycin in a dose of 300 mg in the case of penicillin allergy, every 8 h, ten days before the procedure, and recommend continuing with the same therapy for another seven days after, until the integrity of the mucous membrane is established. In addition, the patient should rinse the oral cavity daily with 10 mL of 0.12% chlorhexidine solution, every 12 h for one minute.

On the other hand, Maxymiw et al. [24] recommend the preoperative administration of penicillin V (phenoxymethylpenicillin) in a dose of 2 g 1 h before the procedure, which, at the same time in a dose of 600 mg four times a day, takes another week after the procedure. Lye et al. [25] prescribed 2 g of penicillin V 1 h before the procedure, which the patient took in combination with metronidazole and with chlorhexidine for another week after the procedure.

#### 2.5.2. Patients Taking Antiresorptive and Antiangiogenic Drugs

There are no universally accepted antibiotic prophylaxis guidelines in patients on antiresorptive and antiangiogenic drugs. Akashi et al. [26] in 2018 published a review paper about use of antibiotics for the prevention of MRONJ, and concluded that oral and maxillofacial surgeons generally prefer penicillin antibiotics with the addition of β-lactamase inhibitors or metronidazole. Therapy duration is individual and based on clinical experience.

Heufelder et al. [27] recommend 875 mg of amoxicillin with 125 mg of clavulanic acid twice a day, or 600 mg of clindamycin three times a day if the patient is allergic to penicillin, 48 h before the procedure and seven days after. In addition, they recommend the perioperative use of topical antimicrobial chlorhexidine mouthwash two to three times daily until mucosal integrity is established.

Recommendations for antibiotic prophylaxis in patients at risk of osteonecrosis of the jaw are summarized in Table 4.

### 2.6. Antibiotic Prophylaxis in HIV-Positive Patients

There is no significant difference in postoperative infections between HIV-infected and HIV-negative patients. Antibiotic prophylaxis should be prescribed individually to each patient in accordance with blood parameter values (number of neutrophils and CD4 + T lymphocytes). If the patient has neutropenia and the number of neutrophils is less than 0.5 × 10^9^/L, AP should be prescribed according to AHA guidelines for the prevention of IE [28].

One should be careful when prescribing antibiotics to HIV-infected patients, especially in the advanced stage of the disease, due to a greater tendency of unwanted reactions such as opportunistic infections and microorganism resistance [29].

### 2.7. Antibiotic Prophylaxis in Patients on Dialysis

There is a low risk of possible infection of the arterio-venous fistula in hemodialysis patients, which can occur during invasive dental procedures, and can result in septicemia, septic emboli, infective endarteritis, and IE [30]. Current AHA guidelines do not include a recommendation of prophylactic antibiotic before invasive dental procedures when performed on patients with intravascular access devices to prevent endarteritis or infective endocarditis, except if the patient has a history of an abscess which was incised and drained [30]. Prophylaxis should be prescribed if the patient has underlying heart disease, for which guidelines recommend antibiotic prophylaxis.

The safest drugs for dialyzed patients are penicillin, clindamycin, and cephalosporins, while tetracyclines and aminoglycosides should be avoided due to their nephrotoxicity.

### 2.8. Antibiotic Prophylaxis in Patients on Biological Therapy

Patients taking biological therapy are at higher risk of infection. Biological therapy is used for autoimmune diseases and cancer, and targets specific parts of the immune system. There is limited information about invasive dental procedures and infection risk in patients taking biological therapy.

For example, according to a patient information leaflet for infliximab (Remicade®, Janssen Biotech, Beerse, Belgium), surgery, dental work, or other invasive procedures should be scheduled between drug infusions. The time that has to pass after the infusion is a minimum of 14 days. The drug should be restarted after the wound from the surgery has healed completely with no sign of infection, and no less than 14 days after the surgery [31].

Since numerous biological drugs are placed on the market every day, and their side effects are recorded, it is necessary to assess the need for prescribing antibiotics before or after an invasive dental procedure individually for each patient.

## 3. Discussion

Antibiotic prophylaxis for infective endocarditis has always been a subject of debate. In 2008, the NICE (National Institute for Health and Care Excellence) published a guideline for antibiotic prophylaxis for patients who are undergoing invasive procedures in dentistry, as well as some non-dental procedures. The aim was to give a recommendation to clinicians, based on the published evidence in the literature. This guideline recommended that patients a risk of infective endocarditis who are undergoing invasive procedures in dentistry, and respiratory, gastrointestinal, or genitourinary invasive procedures, should not take antibiotic prophylaxis for infective endocarditis [32]. After this recommendation, it was shown that infective endocarditis incidence had increased. The reasons were not fully clarified. However, a study in The Lancet from 2015 showed an increase in infective endocarditis cases from 2000 to 2013, following the NICE recommendation [33].

According to the current guidelines, prior to invasive dental procedures, antibiotic prophylaxis is recommended, but less often than in the past. The above is a consequence of spreading awareness of the growing global problem of antimicrobial resistance and a better understanding of the daily incidence of bacteraemia caused by routine activities (chewing, performing oral hygiene). The importance of adequate oral hygiene and regular dental check-ups should be emphasized to all patients. In addition, the antimicrobial effect of antibiotics that are prescribed prophylactically is controversial and a debatable issue regarding the fact that some studies speak in favour of their positive effect, while others do not. According to the latest AHA guidelines from 2021, clindamycin should no longer be prescribed at all as an antibiotic for prophylaxis in the case of hypersensitivity to penicillin due to an increased risk of pseudomembranous colitis. In these situations, cephalexin, doxycycline, azithromycin, or clarithromycin are prescribed [4]. Recently, the European Society of Cardiology published updated guidelines which are in accordance with AHA guidelines, and they no longer recommend clindamycin [12].

Recent meta-analyses have shown that the use of AP is associated with a lower risk of infective endocarditis after invasive dental procedures in patients at high risk. In patients with low or unknown risk, the association was not proven. These results support current AHA and ESC recommendations [34].

Clindamycin is efficient against most odontogenic pathogens, and its use in odontogenic infection is justified, despite not being given for prophylaxis. Regarding adverse reactions, clostridium is associated with diarrhea, on which basis it is excluded from AHA and ESC guidelines; this adverse reaction is associated with all wide-spectrum antibiotics. Therefore, the warning applies to all antibiotics (including clindamycin), which have reported side effects (from mild diarrhea to more severe forms of colitis) associated with *C. difficile* (CDI). These warnings entered the drug information in the period from 2010 to 2014, when they were placed in the ‘Warnings’ section of the drug label. The warnings were subsequently placed in the black box, due to the increasing number of reports of such side effects associated with the use of antibiotics, including clindamycin, in the United States of America. Also, there is a certain group of patients more prone to exhibit this reaction. These are patients older than 65, patients with a previous history of hospitalization, patients frequently receiving antibiotic therapy, and patients on parenteral nutrition. Regarding IE, current guidelines by the AHA and the ESC take into account a narrow spectrum of clinical conditions with a high risk of IE occurrence in which antibiotic prophylaxis should be prescribed before invasive dental procedures.

Guidelines for prescribing antibiotic prophylaxis in patients with implanted artificial joints have been changing from year to year, and included the recommendation of prescribing within the first three months and up to 2 years from the surgical procedure. Back in 2005, there was a protocol in Australia prescribing AP to all patients before tooth extraction within the first three months of artificial joint placement. Five years later, the guidelines were changed due to a higher risk of side effects compared to the benefits derived from AP. Nowadays, in most countries around the world, including the United States, Canada, the United Kingdom, Australia, and New Zealand, the prescription of antibiotic prophylaxis is not recommended for patients with artificial joints [35], even if they are immunocompromised [13,14].

Recent studies also suggest that routine AP before dental procedures is not necessary after total hip or total knee arthroplasty [36], which is very important for reducing unnecessary antibiotic prescriptions, helping reduce antibiotic resistance.

Antibiotic prophylaxis before invasive dental procedures in patients with controlled diabetes is not required. On the other hand, in the case of unregulated disease (where HbA1c is greater than 8%) and if an invasive procedure is necessary, perioperative antibiotic coverage is recommended due to the increased risk of infections, vascular changes, and prolonged wound healing. A full therapeutic dose of antibiotics is usually prescribed, most often penicillin [15].

For patients with transplanted organs, there are no precisely defined guidelines for antibiotic prophylaxis in the pre-transplantation and post-transplantation periods. However, antibiotic prophylaxis is often justified, primarily due to uncontrolled systemic diseases in the pre-transplant period and the increased risk of infections in the post-transplantation period due to high doses of immunosuppressive drugs even though the evidence base is scarce. The American Academy of Pediatric Dentists’ guidelines recommend considering prescribing prophylactic antibiotics according to the AHA guidelines for pediatric dental patients at risk of infection, including transplant recipients on immunosuppressive therapy when the ANC is between 1000 and 2000 cells/mm^3^; when the ANC is <1000, the AAPD recommends antibiotic prophylaxis through an extended course of antibiotics [20,21].

Prescribing antibiotic prophylaxis to patients at risk of osteonecrosis of the jaw is the biggest issue discussed and, accordingly, there are different prescribing protocols depending on the therapist and the clinic. In this group of patients, antibiotic therapy must start a day or a few days before the invasive procedure and continue for some time after it, usually until the integrity of the mucous membrane is restored. The first choice of antibiotic is definitely penicillin, or clindamycin in the case of hypersensitivity to penicillin. Alternatively, a combination of metronidazole and quinolone, or metronidazole and erythromycin, could be prescribed. Certain authors list clindamycin as the first choice of antibiotic primarily because of its broad spectrum and good bone penetration. As it is known for its side effects in patients who are more prone to the development of clostridia-associated enterocolitis, doxycycline could be prescribed six days after the surgery until suture removal [37].

There is a little evidence to support routine antibiotic prophylaxis for all HIV-infected patients. In these patients, it is necessary to seek laboratory parameters (level of neutrophils and CD4 + T lymphocytes), and in case of significant deficiency antibiotic prophylaxis should be prescribed [28,29].

In patients on haemodialysis, the leading cause of death is the occurrence of staphylococcal infection during surgery due to established access to the patient’s bloodstream, with the consequent bacteraemia, which it can lead to. Antibiotic prophylaxis is recommended, according to the latest ADA guidelines, if the patient belongs to the high-risk group of patients for IE due to a certain underlying heart disease [37]. Routine antibiotic prophylaxis is not necessary either in patients on haemodialysis or on peritoneal dialysis [30]. It is possibly considered justified in the first six months from the formed access to the bloodstream in haemodialysis patients [38].

In patients taking biological therapy, the decision to prescribe antibiotics should be made individually. It depends on the patient’s white blood count and the severity of a dental procedure. It is important to do the procedure midway between infusions and not within two weeks before or after receiving the therapy.

## 4. Conclusions and Future Directions

Based on the review of the available literature, it can be concluded that the guidelines and protocols for antibiotic prophylaxis continuously change from year to year based on the knowledge brought by new research. Therefore, the constant education of dentists on the latest guidelines is necessary.

The first choice of antibiotic for antibiotic prophylaxis is always amoxicillin, and, in the case of allergy, azithromycin or clarithromycin should be prescribed. Clindamycin is no longer recommended due to its serious side effect, necrotizing enterocolitis.

In some cases, consultation with a patient’s medical doctor who is in charge of the underlying disease would be necessary to make the right decision for antibiotic prophylaxis. Taking into consideration that each patient needs individual assessment, this interdisciplinary approach is of a great importance for the best outcome of a dental procedure in medically compromised dental patients.

It is also necessary to raise awareness about the unjustified prescription of antibiotics that leads to the increase in the resistance of a large number of microorganisms.

## Figures and Tables

**Table 1:** Diagnoses that require antibiotic prophylaxis before dental procedures [4].

Diagnoses That Requires Antibiotic Prophylaxis Before Dental Procedures [4]
Prosthetic cardiac valves, including transcatheter-implanted prostheses and homografts
Prosthetic material used for cardiac valve repair, such as annuloplasty rings, chords or clips
History of previous IE
Unrepaired cyanotic congenital heart defect or repaired congenital heart defect, with residual shunts or valvular regurgitation at the site of or adjacent to the site of a prosthetic patch or prosthetic device *
Patients with cardiac transplant with valve regurgitation due to a structurally abnormal valve

* Except for the listed diagnoses, antibiotic prophylaxis prior to invasive dental procedures is not recommended for other types of congenital heart defect.

**Table 2:** Recommendations for antibiotic regimen in patients with diabetes undergoing dental procedures [16].

Fasting Plasma Glucose Level	Antibiotic Regimen
<200 mg/dL	Generally not required
>200 mL/dL	Prophylaxis prior to dental procedure is not necessary but antibiotics should be given postoperatively in case of invasive dental procedures

**Table 3:** Recommendations for dental treatment in patients with transplanted organs [18,19,20,21].

Recommendations for Dental Treatment in Patients with Transplanted Organs [18,19,20,21]	
Pre-transplantation period	Remove all potential source of infection. No general recommendation for AP but can be justified if patients have an uncontrolled systemic disease such as uncontrolled diabetes, high-risk cardiovascular diseases, blood dyscrasias, and others. Check complete blood count, coagulation test, and others, depending on the underlying disease. Consultation with the patient’s doctor is essential.
Post-transplantation period	Prescribe AP due to immunosuppression and high risk of infection. If ANC is between 1000 and 2000 cells/mm^3^, consider AP according to the AHA guidelines. If ANC is less than 1000 cells/mm^3^, AP and extended course of antibiotic should be prescribed.

**Table 4:** Recommendations for antibiotic regimen in patients at risk of osteonecrosis of the jaw.

Recommendations for Antibiotic Regimen in Patients with at the Risk for of Osteonecrosis of the Jaw	
Patients with head and neck irradiation	No universally accepted guidelines exist! A total of 500 mg amoxicillin or 300 mg clindamycin in the case of an allergy to penicillin—every 8 h, 10 days before, and continue for 7 days after the procedure [23]. Penicillin V (phenoxymethylpenicillin)—2 g 1 h before the procedure and a dose of 600 mg four times a day another week after the procedure [24]. Penicillin V 2g 1 h before the procedure, which the patient takes in combination with metronidazole and with chlorhexidine for another week after the procedure [25].
Patients taking antiresorptive and antiangiogenic drugs	No universally accepted guidelines exist! A total of 875 mg of amoxicillin with 125 mg of clavulanic acid twice a day, or 600 mg of clindamycin three times a day if the patient is allergic to penicillin, 48 h before and seven days after the procedure with chlorhexidine mouthwash, 2–3 times daily [27].
